# Intestinal metastases diffused from primary gastric adenocarcinoma with signet ring cells: case report

**DOI:** 10.3389/fonc.2026.1859707

**Published:** 2026-07-13

**Authors:** Haiping Qi, Weiyan Li, Xingting Luo, Bin Huang, Xiaoguang Shi, Xuhua Xiao

**Affiliations:** Department of Gastroenterology, The First Affiliated Hospital of Guilin Medical University, Guilin, China

**Keywords:** CK7/CK20 immunohistochemistry, gastric carcinoma, peritoneal seeding, polypoid intestinal metastases, signet ring cell carcinoma

## Abstract

Signet ring cell carcinoma (SRCC), a poorly differentiated subtype of gastric adenocarcinoma, is known for its high metastatic capacity. However, metastases to the intestines, both small and large, are uncommon. This report describes a unique case of gastric adenocarcinoma with signet ring cells features and multiple polypoid intestinal metastases.

**Case presentation:**

A 62-year-old man with primary gastric SRCC exhibited epigastric pain, lower abdominal distension, and a significantly elevated serum CEA level (400 ng/ml). Endoscopic evaluations detected numerous ulcerative and polypoid growths in the stomach, duodenum, distal ileum, ileocecal valve, and colorectum. Imaging studies confirmed thickening of the gastric wall, abdominal lymph node enlargement, and multiple bone metastases. Examination of tissue samples using histopathological and immunohistochemical methods indicated poorly differentiated adenocarcinoma with signet ring cell features, confirming the intestinal lesions as metastases of gastric SRCC.

**Conclusion:**

In this report, we describe a rare instance of gastric adenocarcinoma characterized by signet ring cells and multiple intestinal polypoid metastases. Differentiating between metastatic gastric signet ring cell carcinoma (SRCC) and primary colorectal carcinoma can be aided by assessing CK7 and CK20 expression, where CK7+/CK20+ is more frequently observed in gastric adenocarcinomas. The precise mechanism underlying this distinctive metastatic pattern remains uncertain, although peritoneal seeding has been proposed as a potential route. Treatment protocols for such cases are not standardized; typically, systemic chemotherapy is favored over surgical intervention for widespread lesions, and the prognosis is generally unfavorable. This case underscores the unusual metastatic patterns of gastric SRCC, emphasizing the importance of thorough endoscopic and pathological assessments to prevent misdiagnosis and offering clinical guidance for managing uncommon metastases in gastric cancer.

## Background

Gastric carcinoma is the fifth most frequent cancer in the world. Signet ring cell carcinoma (SRCC) is a well-known poorly differentiated histological variant of gastric cancer with a significant metastatic tendency. Hematogenous spread, lymphatic metastases, direct local invasion of nearby organs, and peritoneal seeding or transcoelomic spread are the three main ways that gastric cancer spreads. Additionally, the most prevalent path is lymphatic metastases. The liver, lungs, bones, and adrenal gland are the main sites of hematogenous spread. However, colorectal along with small intestinal metastases are extremely rare. We present a case of gastric carcinoma characterized by symptoms of stomach pain and an elevated serum level of carcinoembryonic antigen (CEA). The pathological diagnosis was established through histological examination of endoscopic biopsy specimens. This report details the endoscopic features of small intestinal and colorectal metastases, which manifested as multiple polyps and superficial ulcerative lesions originating from primary gastric signet-ring cell carcinoma (SRCC).

## Case presentation

**Figure 1:**
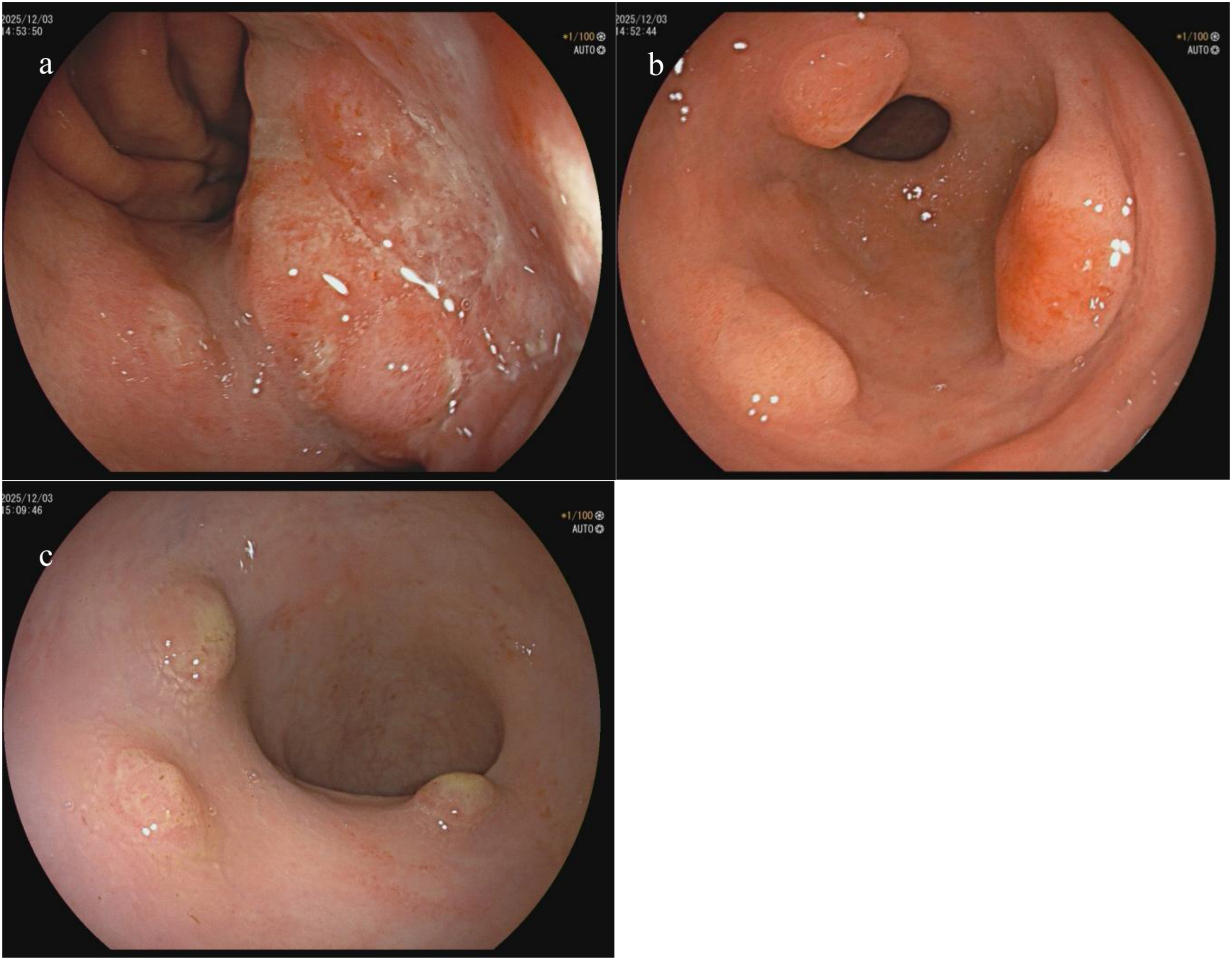
An irregularly large depressed lesion in the gastric angle **(A)** and esophagogastroduodenoscopy images showing multiple “polyps” in the gastric antrum **(B)**;colonoscopy images showing multiple “polyps” throughout colorectum **(C)**.

**Figure 2:**
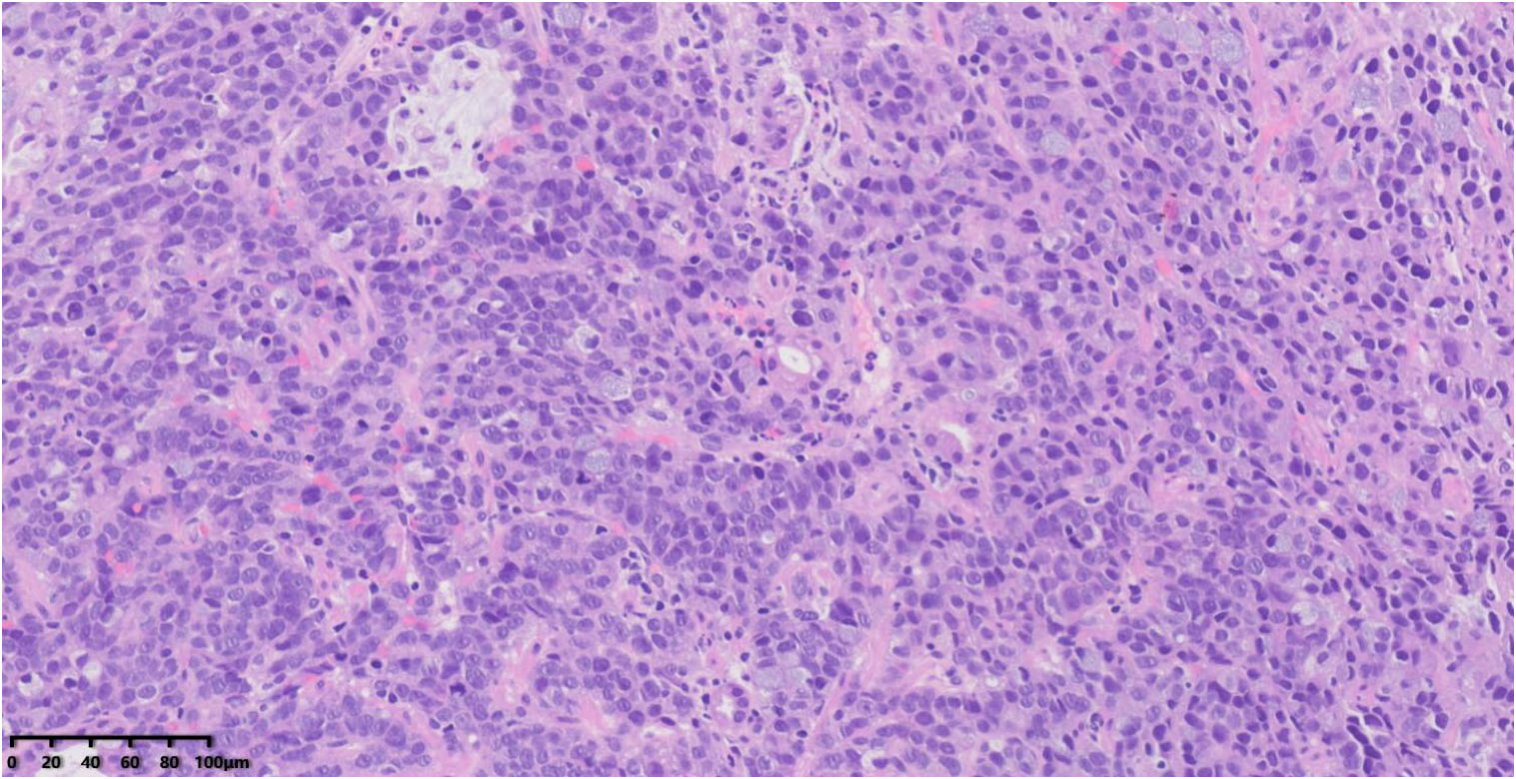
Histopathological examination result revealing poorly differentiated adenocarcinoma and signet ring cells (hematoxylin and eosin staining).

A 62-year-old man presented with epigastric pain persisting for the past year and lower abdominal fullness for two weeks. There was a history of hypertension in the past, and blood pressure was well controlled. On November 23, 2025, he underwent esophagogastroduodenoscopy and colonoscopy at a local hospital, which revealed a large ulcerative lesion in the gastric angle and multiple ulcerative lesions in the gastric antrum. Biopsy of the lesions indicated adenocarcinoma. Total colonoscopy identified numerous polypoid lesions in the descending colon and rectum, measuring 10–12 mm in diameter, as well as an ulcerative lesion in the transverse colon.The physician performed an endoscopic mucosal resection (EMR), which revealed that all lesions were consistent with adenocarcinoma. Consequently, the patient was referred to my hospital for advanced treatment. The serum level of CEA was elevated at 400 ng/ml, CA199 7.29ng/ml, CA153 4.11ng/ml. An abdominal computed tomography (CT) scan with intravenous contrast was conducted, revealing diffuse, uneven thickening of the gastric antrum accompanied by peripheral lymphadenopathy, indicative of gastric carcinoma. Additionally, single photon emission computed tomography (SPET-CT) demonstrated multiple bone metastases.We obtained the patient’s consent and subsequently conducted a repeat esophagogastroduodenoscopy and colonoscopy. The esophagogastroduodenoscopy revealed an irregularly large depressed lesion in the gastric angle, which involved the greater curvature of the gastric body and the gastric antrum, as well as numerous flat elevated lesions in the gastric antrum and duodenum. Total colonoscopy identified multiple polypoid lesions in the ascending, transverse, sigmoid, and rectal colon, ranging 8 to 12 mm in diameter, along with erosions in the distal ileum and superficial ulcerative lesions in the ileocecal valve and transverse colon. These lesions exhibited well-defined margins and depressions at their tips (**Figures 1A–C**). The pathological report, which arrived several days later, was unexpected and indicated the presence of nine lesions in the specimen. All biopsy specimens were confirmed to be poorly differentiated adenocarcinoma with signet ring-cell differentiation (**Figure 2**), affecting the stomach, duodenum, distal ileum, ileocecal valve, and colorectum. Immunohistochemical staining results showed CK (+), CD56 (-), Her-2 (0), P53 (+, wild type), Ki67 index: 70%, CK7 (+), CK20 (part +), Villin (+), Claudin18.2 (2+/3+, approximately 50%), MSH2 (+), MSH6 (+), MLH1 (+), and PMS2 (+). Given that all biopsy specimens exhibited a definitive morphological diagnosis (poorly differentiated adenocarcinoma with signet ring cell differentiation) on H&E staining, MUC family mucin markers (such as MUC2 and MUC5AC) were not included in the testing panel. The gastric, small intestinal, and colorectal biopsy specimens exhibited identical manifestations in both microscopy and immunohistochemical staining. We conclude that this represents a rare case of gastric signet ring-cell adenocarcinoma metastasizing to the small intestine and colorectum, presenting as flat elevated lesions and ulcerative lesions, accompanied by extensive lymphatic spread to the abdomen and osseous metastasis, without evidence of visceral metastasis. The patient declined comprehensive genetic testing due to financial concerns and reported no family history of tumors. Ultimately, he underwent one cycle of chemotherapy with tislelizumab and oxaliplatin, which was complicated by severe chemotherapy-induced bone marrow suppression. Subsequently, the patient refused any further systemic chemotherapy.

## Discussion and conclusion

Poorly cohesive carcinoma, known as signet ring cell carcinoma (SRCC), is characterized by tumor cells with abundant cytoplasmic mucin and a crescent-shaped nucleus. Despite the decline in gastric cancer cases globally due to effective Helicobacter pylori treatment, SRCC rates are increasing, representing 8–30% of gastric cancers (1).Clinically, SRCC poses significant challenges as it is frequently detected at advanced stages in younger individuals, leading to a dismal prognosis.

Signet ring cell adenocarcinomas in the colon are rare, with fewer cases documented in the PubMed database (2). Small intestinal metastasis has not been observed thus far. Differentiating colonic metastasis from normal polyps and primary colonic carcinoma can be challenging. Zhang et al. (2) identified key distinguishing factors: a history of primary gastric SRCC, distinct colonoscopy findings such as unclear margins, erosion, or depression on metastatic polyps, the tendency for metastatic lesions to be multiple, discrete, and polymorphic, and the typical pathological features of SRCC under microscopy.A critical differential diagnostic question in this case is whether the multiple intestinal lesions represent metastases from gastric cancer or synchronous multicentric primary tumors. We support the former interpretation based on the following evidence. First, histomorphological consistency. All biopsy specimens from the stomach, duodenum, distal ileum, ileocecal valve, and colorectum exhibited identical morphological features—poorly differentiated adenocarcinoma with signet ring cell differentiation—without site-specific variations. This homogeneous morphology across all sites strongly favors a common origin. Second, clinical stage context. At presentation, the patient was at a widely disseminated stage, with markedly elevated serum CEA (400 ng/ml), abdominal CT showing gastric antrum thickening with peripheral lymphadenopathy, and SPET-CT demonstrating multiple bone metastases. In the presence of established hematogenous (bone) and lymphatic (abdominal lymph nodes) metastases, the multiple intestinal lesions are more consistent with metastatic dissemination than with synchronous development of multiple primary carcinomas. Third, immunohistochemical analyses are conducted to distinguish between primary colon and metastatic gastric cancer in cases of signet ring cell adenocarcinoma diagnosed during colonoscopy. Tumor markers CK7 and CK20 are commonly utilized for this purpose. CK7 is typically expressed in most carcinoma cases, except for those originating from the prostate, colon, thymus, and kidney, as well as in carcinoid tumors from the lungs and gastrointestinal tract, and Merkel cell tumors of the skin. On the other hand, CK20-positive staining is commonly observed in nearly all colorectal carcinoma and Merkel cell tumor cases, as well as in a significant proportion of patients with pancreatic carcinoma (62%), gastric carcinoma (50%), cholangiocarcinoma (43%), and transitional cell carcinoma (29%) (3). A hypothesis suggests that the presence of a CK7-/CK20+ staining pattern in neoplastic cells supports a colonic origin when a signet ring cell adenocarcinoma is detected on colon biopsy, whereas a CK7+/CK20 staining pattern indicates a gastric origin (4). However, Chu et al. (5) found that 13% (1/8) of gastric carcinomas and 5% (1/20) of colorectal carcinomas exhibited a CK7+/CK20+ pattern. Similarly, Maral et al. (6) observed that carcinomas of upper gastrointestinal tract 10.25% and carcinomas of lower gastrointestinal tract 7.69% were CK7+/CK20+. Therefore, the CK7+/CK20+ staining pattern is more prevalent in gastric adenocarcinomas than in colorectal cancer. In this case, the biopsy samples showed positive staining for both CK7 and CK20. Admittedly, the absence of MUC2 and MUC5AC staining limits further phenotypic subclassification, which constitutes a limitation of this study. Nevertheless, based on the integration of gastroenterological endoscop, morphological and clinico-radiological evidence, we have sufficient grounds to classify the intestinal lesions as metastases from gastric cancer.

Metastasis of gastric carcinoma to the small bowel and colon can manifest as either solitary or multiple colonic polyps. This rare condition has not been documented in the literature before. Gastric or gastric stump carcinoma can metastasize to the colon, presenting as solitary or multiple colonic polyps. The first reported case was by Metayer et al. (7) in 1991, followed by Ogiwara et al. (8) in 1994. In complex scenarios like these, a differential diagnosis is necessary. A confirmed history of gastric SRCC, typical colonoscopic appearance, and distinct pathological findings can help establish the link between colonic metastases and the primary gastric tumor.The specific mechanism of this unique form of metastasis is still not understood. Intestinal metastases are commonly linked with peritoneal seeding, with liver involvement being rare. The primary mode of secondary neoplastic infiltration in the bowel is peritoneal seeding, typically stemming from ovarian cancer. Hematogenous spread is infrequent in cases affecting the small bowel (9); when it does occur, the primary tumor usually originates from breast cancer (10, 11), lung cancer, or melanoma (12). Colonic metastases are uncommon and usually originate from carcinoma of the breast (13), stomach (3), skin (melanomas), kidney (14, 15), prostate (16), or ovary (17), pancreas (18). Colonic metastases in a polypoid form commonly arise from melanoma (19) or spindle-cell renal carcinoma (20). Treatment strategy remains undefined due to a scarcity of cases. Gao et al. (3) noted in their research that these nodules may distribute across the colon and rectum. In such instances, systemic chemotherapy could be favored over surgical intervention. Irrespective of the therapeutic approach chosen, this condition signifies an unfavorable outlook, culminating in rapid fatality. In summary, we described a rare metastatic pattern in primary gastric SRCC, spreading to the small bowel and colorectum, manifesting as multiple flat elevated polypoid lesions in these regions. The existence of multiple polypoid metastases in the small bowel and colorectum may indicate an exceptionally uncommon manifestation of widespread SRCC dissemination. When multiple signet ring cell adenocarcinomas are found in the stomach, it should be considered that there may be metastasis throughout the entire gastrointestinal tract. In addition to gastroscopy, colonoscopy should also be completed.

## Data Availability

The original contributions presented in the study are included in the article/supplementary material. Further inquiries can be directed to the corresponding author.
